# Hyperkalemia Following Parathyroidectomy in Patients with Renal Hyperparathyroidism—New Thresholds for Urgent Perioperative Dialysis

**DOI:** 10.3390/jcm11020409

**Published:** 2022-01-14

**Authors:** Claudia Bures, Yasmin Uluk, Mona Besmens, Aycan Akca, Eva-Maria Dobrindt, Johann Pratschke, Peter Goretzki, Martina Mogl, Deniz Uluk

**Affiliations:** 1 Department of Surgery, Campus Mitte, Campus Virchow-Klinikum, Charité-Universitätsmedizin Berlin, 10117 Berlin, Germany; claudia.bures@charite.de (C.B.); yasmin.uluk@googlemail.com (Y.U.); eva-maria.dobrindt@charite.de (E.-M.D.); johann.pratschke@charite.de (J.P.); peter.goretzki@charite.de (P.G.); martina.mogl@charite.de (M.M.); 2 Department of Surgery, Rheinland Klinikum Lukaskrankenhaus Neuss, 41464 Neuss, Germany; mona.besmens@rheinlandklinikum.de (M.B.); aakca@lukasneuss.de (A.A.)

**Keywords:** secondary hyperparathyroidism, perioperative hyperkalemia, parathyroidectomy, urgent dialysis

## Abstract

Parathyroidectomy (PTX) is a mainstay of treating secondary hyperparathyroidism (SHPT) in patients with kidney failure in order to reduce the incidence of cardiovascular events (CVE), increase overall survival and improve quality of life. Perioperative hyperkalemia may lead to devastating cardiac complications. Distinct preoperative thresholds for serum potassium levels (SPL) were defined, but neither their usefulness nor consecutive risks are understood. This study compared the results and efficacy of different clinical procedures in preventing or treating perioperative hyperkalemia, including postoperative urgent hemodialysis (UHD). *Methods:* Patients from Charité-Universitätsmedizin Berlin and Rheinland Klinikum Lukaskrankenhaus, Neuss, undergoing PTX due to SHPT between 2008 and 2018 were analyzed retrospectively with regard to demographic parameters, surgery specific conditions and perioperative laboratory results. Comparisons of patient values from both centers with focus on perioperative hyperkalemia and the need for UHD were performed. *Results*: A total of 251 patients undergoing PTX for SHPT were included (Neuss: *n* = 121 (48%); Berlin: *n* = 130 (52%)). Perioperative hyperkalemia (SPL ≥ 5.5 mmol/L) was noted in 134 patients (53%). UHD on the day of surgery was performed especially in patients with intraoperative hyperkalemia, in females (*n* = 40 (16%) vs. *n* = 27 (11%); *p* = 0.023), in obese patients (*n* = 27 (40%) vs. *n* = 50 (28%), *p* = 0.040) and more often in patients treated in Neuss (*n* = 42 (35%) vs. 25 (19%); *p* = 0.006). For patients treated in Neuss, the intraoperative hyperkalemia cut-off level above 5.75 mmol/L was the most predictive factor for UHD (*n* = 30 (71%) vs. *n* = 8 (10%); *p* < 0.001). Concerning secondary effects of hyperkalemia or UHD, no patient died within the postoperative period, and only three patients suffered from acute CVE, with SPL > 5.5 mmol/L measured in only one patient. *Conclusion:* Perioperative values could not predict postoperative hyperkalemia with the need for UHD. Previously defined cut-off levels for SPL should be reconsidered, especially for patients undergoing PTX. Early postoperative dialysis in patients with postoperative hyperkalemia can be performed with a low risk for complications and may be indicated for all patients with increased perioperative SPL.

## 1. Introduction

Secondary hyperparathyroidism (SHPT) is a common and serious sequela of end-stage renal disease (ESRD), requiring surgical parathyroidectomy (PTX) if medical treatment (i.e., calcimimetics and vitamin D analogs) fails [1,2,3,4,5]. PTX is required in 15% of patients after ten years of renal replacement therapy and even in 38% after 20 years of ongoing hemodialysis (HD) [6]. According to the Kidney Disease Outcomes Quality Initiative (KDOQI), PTX is recommended in patients with chronic kidney disease (CKD) stages 3–5, parathyroid hormone levels (PTH) beyond 800 ng/mL and no response to medical therapy [5]. The benefits of PTX have been well known since 1965 and were first described by R.E. Wilson and coworkers [7]. PTX restores physiologic calcium and phosphate hemostasis and normalizes PTH levels and bone metabolism [7,8]. These benefits of PTX are based on observational studies, not on randomized clinical trials describing the probable beneficial effects of surgery on both short-term laboratory parameters and long-term clinical outcomes such as osteoporotic fractures or increased mortality due to cardiovascular events (CVE) [9,10]. Altogether, PTX increases quality of life and even life expectancy [1,11,12,13]. Patients with ESRD have a markedly increased incidence and mortality of CVE compared with the age-matched general population [1,2,14,15]. In some reports early postoperative deaths have been reported in patients with SHPT and were attributed to changes in perioperative electrolytes, especially serum potassium levels (SPL) [13,16,17]. While previous observational studies have associated hyperkalemia with a higher mortality in patients after PTX, various reasons for and definitions of hyperkalemia were applied. Critically high SPL affecting cardiac function is hypothesized to be between 5.5 and 6.0 mmol/L [17,18,19,20]. Hyperkalemia affects 25–80% of patients receiving HD during the first 24 h after PTX [21,22,23,24] and seems to occur more frequently in patients with SHPT after surgical treatment compared with medical treatment alone [25,26]. An increase in SPL was associated with perioperative hypocalcemia after successful PTX, as reported by some authors [23,26,27]. Previous studies recommended to cancel surgery or to perform urgent hemodialysis (UHD) if SPL was elevated above 5.3–5.5 mmol/L in patients preoperatively [22,28]. For this reason, the perioperative management of SPL is crucial in patients with ESRD undergoing PTX. This observational study investigated the impact of increased SPL during or immediately after PTX and the different clinical procedures of two high-volume endocrine surgery departments in preventing adverse effects of hyperkalemia and UHD on the day of surgery.

## 2. Methods

### 2.1. Data Collection

Patients with secondary hyperparathyroidism (SHPT) and concomitant end-stage renal disease (ESRD) undergoing parathyroidectomy (PTX) between 2008 and 2018 at the endocrine surgical departments of Charité-Universitätsmedizin Berlin and Rheinland Klinikum Lukaskrankenhaus, Neuss, Germany, were included. All data were acquired retrospectively from the electronic medical system. The operative procedures were performed or supervised by surgeons specialized in endocrine surgery according to departments’ standards following a comparable algorithm. Data collection included demographic parameters such as gender, age, comorbidities, current medication, reasons for dialysis and time of dialysis-dependency. Standard operating procedures (SOP) of both centers contained oral calcium hemostasis-regulating medication (i.e., calcium, calcimimetics, vitamin D) to prevent hungry bone syndrome. Surgery-specific values such as duration of surgery, performed procedure and number of resected parathyroid glands were included. A body mass index (BMI) ≥ 30 kg/m^2^ was defined as obesity due to actual consensus of the World Health Organization (WHO) [29]. Development of pre-, intra- and postoperative laboratory values for serum potassium (SPL), serum calcium (SCL) and parathyroid hormone (PTH) level were recorded. Preoperative blood draws were taken on the morning before surgery; postoperative blood draws were taken immediately after PTX at the anesthesiologic recovery room or at the post-anesthesia care unit (PACU). Hyperkalemia was defined as a SPL ≥ 5.5 mmol/L. Patients in need of urgent hemodialysis (UHD) on operation day were categorized into group A and received renal replacement therapy (RRT) for up to 4 h with a potassium concentration of 2–3mmol/L in the dialysate, depending to the patient’s baseline SPL, until a tolerable value was reached immediately after surgery. The remaining patients without the need for UHD were summarized into group B. All patients received hemodialysis because of ESRD and perioperative protocol for both centers scheduled dialyses on the day before surgery either in-hospital or at the patient’s regular dialysis center.

### 2.2. Privacy and Ethics

Compliance with ethical aspects and data protection regulations were ensured. All procedures performed were in accordance with the 1964 Helsinki Declaration and its later amendments. An ethical approval for processing and publication of the data was obtained from the institutional ethics committee (Charité-Universitätsmedizin Berlin, Germany, EA4/086/19).

### 2.3. Statistical Analysis

Data collection and statistical analysis were performed with IBM SPSS Statistics (IBM SPSS^®^ 25.0, Armonk, NY, USA). Patients were analyzed for risk factors for perioperative hyperkalemia and the need for UHD on the day of surgery. Descriptive values are shown as absolute values and percentages, in mean and standard deviation in normally distributed parameters and in median and range in the case of a non-normal distribution. In the case of Gauss’ distribution, Student’s t-test was performed to evaluate statistical significance, and Mann–Whitney U-Test was performed for non-normal distributed quantitative variables. Categorial data were tested by chi-squared test and Fisher’s exact test. The receiver operating characteristic curve (ROC) method was used to assess the cut-off point for probable prediction of perioperative SPL concerning hyperkalemia and UHD. Binary logistic regression was performed according to SPL and SCL perioperatively to determine the effects of rising SPL with regard to the risk for UHD or appearance of hyperkalemia. The statistical significance level was set at *p*-value < 0.05.

## 3. Results

Two hundred and fifty-one patients were included in this study, with comparable numbers between both centers (Table 1). The reasons for ESRD differed between patients from both centers, as shown in Table 1. Comparing comorbidities, obesity was more frequent in patients from Neuss (52% vs. 11%, *p* < 0.001). Cinacalcet as primary medical treatment of secondary hyperparathyroidism (SHPT) was prescribed more often in Neuss than in Berlin. Mean preoperative serum calcium levels (SCL) were 2.3 mmol/L and 2.4 mmol/L; parathyroid hormone levels (PTH) were 612 ng/L and 1105 ng/L in patients from Berlin and Neuss, respectively (Table 2). Preoperative hyperkalemia was noted more often in patients from Neuss (*n* = 40 (33%) than in patients from Berlin (*n* = 30 (23%), *p* > 0.001). Urgent hemodialysis (UHD) was performed significantly more often in Neuss compared with Berlin on the day of parathyroid surgery (*n* = 42 (35%) vs. *n* = 25 (19%); *p* = 0.006).

Altogether, 63 patients (25%) developed an intra- or postoperative elevation of SPL above 5.5 mmol/L during or after parathyroidectomy (PTX). Sixty-seven patients (27%) underwent UHD immediately on the day of PTX. UHD was significantly associated with intra- and postoperative hyperkalemia, as shown in Table 3 (*p* < 0.001). Preoperative hyperkalemia was measured in approximately one-third of all patients (28%), with no impact on the need for UHD (*n* = 19 (28%) vs. 51 (28%); *p* = 0.984). Comparing both centers, intra- and postoperative hyperkalemia was detected more frequently in patients from Neuss (*p* < 0.001), and UHD was performed more often in patients from Neuss (*p* = 0.009) (Table 2). Additionally, female gender and obesity demonstrated a statistically significant association with a higher incidence of UHD, whereas diabetes mellitus, CVD, preoperative Cinacalcet therapy and time on dialysis did not (Table 3).

Since UHD showed significant differences between both centers, logistic regression analysis concerning the impact of hyperkalemia demonstrated a 3-fold increase in the odds for UHD in patients with elevated SPL (OR 3.2, CI-95%: 2.1–4.9, *p* < 0.001). For the whole study group, intraoperative elevation of SPL above 5.5 mmol/L even had a 6-fold increase in the odds for UHD (OR 6.0, 95%CI: 1.9–18.9; *p* = 0.001). 

Regarding parathyroid surgery, subtotal parathyroidectomy was the preferred surgical procedure in both centers (Table 2), but selective PTX was performed more often in Berlin (*n* = 13 (10%) vs. *n* = 6 (5%)), while total PTX was performed more often in Neuss (*n* = 44 (36%) vs. *n* = 9 (22%), *p* = 0.028). Duration of surgery was significantly shorter in Neuss than in Berlin (*p* = 0.010) (Table 2). Neither the surgical procedure nor the duration of surgery significantly affected postoperative increase in SPL (*p* = 0.987) or the need for UHD (*p* = 0.921), respectively. 

Median preoperative PTH levels were 849 ng/L (85–3108 ng/L), with a postoperative decrease to a median level of 15 ng/L (3–1019 ng/L). There was no correlation between preoperative PTH levels, decrease in elevated PTH levels and UHD. 

As demonstrated in Table 1, sex was evenly distributed in all patients, while more female patients underwent UHD and showed an approximately two-fold increase in the odds for UHD (OR 1.9, CI-95%: 1.1–3.4; *p* = 0.023) (Table 3). Obesity was seen more often in patients with UHD (*n* = 27 (40%) vs. *n* = 50 (28%); *p* = 0.040), while more patients treated in Neuss were obese.

Intra- and postoperatively elevated SPL were associated with increased UHD events. Mean intraoperative SPL was 5.6 ± 1.0 mmol/L in the UHD group compared with 4.8 ± 0.7 mmol/L in the control group (*p* < 0.001). An intraoperative increase in SPL was associated with a 3-fold increase in the odds for UHD in Neuss (OR 3.8, CI-95%: 2.0–7.1; *p* < 0.001). Patients who had UHD on the day of surgery had a statistically significant increase in SPL by approximately 8% when compared with patients not receiving UHD on the day of surgery (0.02%, *p* = 0.005). The development of perioperative SPL is demonstrated in Table 4, and its center-specific association with UHD is shown in Figure S1. In Neuss, postoperative hyperkalemia occurred in 81% of patients receiving UHD, and the average SPL increased by 16% from the preoperative value (*p* < 0.001) (Supplementary Material Table S1). Receiver operating characteristics (ROC) analysis to determine a probable predictive SPL for UHD showed fair results for an intraoperative SPL of 5.75 mmol/L (Supplementary Materials Figure S2, AUC = 0.725; *p* < 0.001).

Intraoperative hyperkalemia was significantly associated with the lowest measured calcium values (1.76 ± 0.29 mmol/L vs. 1.86 ± 0.29 mmol/L; *p* = 0.014) and with a perioperative calcium decrease (26% ± 11% vs. 20% ± 13%; *p* = 0.003). A decrease in serum calcium levels (SCL) after PTX was associated with a 5-fold increase in the odds for intraoperative hyperkalemia (OR 5.3, CI-95%: 1.8–15.4; *p* = 0.002) and a 4-fold increase in the odds for UHD (OR 4.3, CI-95%: 1.5–11.9; *p* = 0.005).

Perioperative CVD events were detected in only three patients within the whole study cohort. A 52-year-old male patient in Neuss suffered from retrosternal pain and shortness of breath immediately after surgery. This patient’s preoperative SPL was 6.9 mmol/L and decreased to 6.25 mmol/L postoperatively. He received UHD immediately after PTX. A serum troponin increase up to 100 pg/mL and new negative T-waves were detected in the ECG. Coronary angiography confirmed a NSTEMI, and the patient was treated by angioplasty of the right coronary artery. In Berlin, a 65-year-old female patient suffering from new onset tachyarrhythmia was treated with antiarrhythmic medication and left the ICU after one day, while the surgery of a 37-year-old man was interrupted by a cardiogenic shock at the beginning of the anesthesia. Further investigations including coronary angiography ruled out any cardiovascular pathology leading to the assumption of an anaphylactic reaction. No patient died during the hospitalization.

## 4. Discussion

This study investigated the occurrence of hyperkalemia during and immediately after parathyroidectomy (PTX) in patients with end-stage renal disease (ESRD) and the feasibility of urgent hemodialysis (UHD) on the day of surgery. Female gender and obesity tended to be demographic parameters, while perioperative serum calcium levels (SCL) and its decrease could predict intraoperative hyperkalemia the most. Serum potassium levels (SPL) measured intraoperatively above 5.75 mmol/L were evaluated as a potential cut-off value in predicting mandatory dialyses on the day of surgery. Neither hyperkalemia nor UHD caused severe complications in this investigation comparing two large series of patients with secondary hyperparathyroidism (SHPT).

PTX is the standard of care for SHPT in patients with ESRD after failure of medical treatment [5,30], but previous studies reported potentially life-threatening hyperkalemia as a significant risk perioperatively [31]. While dialysis is indicated for SPL > 5.5 mmol/L within elective procedures at many centers, in this investigation two different strategies were observed handling perioperative hyperkalemia. Hyperkalemia already occurred in approximately one-third of the cohort (28%) preoperatively and could be detected during the whole perioperative period in up to 53% of patients after PTX without any clinical consequences. Comparing the centers and the groups, there were an increased number of patients suffering from hyperkalemia in Neuss and in patients receiving UHD (group A). However, UHD was mainly performed at Neuss and was predicted significantly by intraoperative hyperkalemia. Previous investigations demonstrated the impact of decreasing SCL immediately after PTX due to rapid loss of PTH [8,23,26,27]. The sharp decrease in extracellular calcium caused by sudden bone absorption by enhanced osteoblast activity and simultaneously inhibited osteoclast activity may result in a dysregulation of the membrane’s sodium–potassium pump by adversely increased activity of the calcium–sodium carrier (Figure S3) [17,23,26,32,33,34]. Further, PTH may enhance the effect of increased SPL by impaired extrarenal disposal of potassium in the tissue in anuric patients [34,35]. Timofte et al. consequently mention the effect of inadequate nutrition of patients with ESRD due to the impact of reduced insulin secretion and therefore less cellular potassium uptake, which also may play a decisive role in fasting patients undergoing surgery, causing hyperkalemia [32]. These circumstances are hypothesized to cause rising SPL and hyperkalemia. Perioperative decrease in SCL was significantly associated with rising SPL and the occurrence of intraoperative hyperkalemia. Chong et al. stated that with the first resected parathyroid gland, hyperkalemia already appears and continues with every further removed gland [25]. In Neuss, total PTX was performed the most and may have encouraged this correlation of removed glands, resulting in hypocalcemia and consecutive intraoperative hyperkalemia. Although, both centers pursued the target of preventing hungry bone syndrome by sudden SPL decline after abrupt missing of PTH with their perioperative SOPs through sufficient calcium supply and vitamin D medication, there were marginal but significant differences in SCL between both places.

Song et al. mentioned preoperative SPL as an independent risk factor for postoperative hyperkalemia and identified a SPL of 3.9 mmol/L as a predictive value preoperatively [23]. Zou et al. recommended a preoperative SPL below 4.3 mmol/L to prevent postoperative hyperkalemia [26]. Preoperative SPL showed no significant influence on hyperkalemia or the need for UHD on the day of surgery in this investigation. Instead, the increase in SPL and the appearance of hyperkalemia intraoperatively increased the need for UHD. Intraoperative hyperkalemia showed significant associations with decreased SCL. Interestingly, the perioperative SPL increased in Neuss, while in Berlin, SPL decreased, and thus, UHD on the day of surgery was performed rarely in Berlin. Comparing both centers, the anesthesiologic regimen seemed to have a major influence on the development of SPL, e.g., the use of crystalloid solutions with less potassium or dextrose-containing solutions with insulin resulted in serum electrolyte changes. This perception may allow a new perspective on the application of hemodialysis on the day of surgery and may change a surgeon’s behavior concerning perioperative hyperkalemia and the cancelation of procedures.

Previous analysis showed an increased incidence of postoperative hyperkalemia in younger and male patients [31,36,37]. In this investigation, female gender showed an approximately 2-fold higher risk in the odds for urgent dialysis on the day of surgery (OR 1.9; CI 95%: 1.1–3.4; *p* = 0.023). Considering the influence of demographic parameters, only obesity was not evenly distributed between the groups. While patients from one center were significantly more obese, patients with the need for UHD were also significantly more obese in the whole cohort. Previous studies analyzing the association of obesity and perioperative electrolyte changes in patients with SHPT are sparse. Fotheringham and coworkers (2014) did not confirm these findings; instead, they stated an association of lower odds for hyperkalemia in obese men [38]. The duration of surgery and its relevance concerning the elevation of SPL have been investigated previously, describing an increase in SPL during prolonged procedures [24,31]. These findings were not confirmed in this investigation.

Several studies have shown that preoperative SPL independently influences postoperative concentrations [17,22,36,37,38]. This investigation, however, demonstrated the major influence of perioperative sharp decrease in SCL, increased intraoperative SPL above 5.75 mmol/L and its impact on UHD on the day of surgery. 

Cardiovascular events (CVE), such as tachyarrhythmias or myocardial infarction, as perioperative complications in patients with ESRD undergoing PTX, are described in previous investigations and are feared similarly by anesthesiologists and surgeons [14,16,17]. Although hyperkalemia is a well-known risk factor for severe CVE, only one patient in this observational study suffered from a postoperative NSTEMI and simultaneous elevated SPL. 

The limitations of this observational study are foremost its retrospective nature. Furthermore, the impact of several parameters such as alkaline phosphatase or phosphate itself were not part of the analyses. Standardized electrocardiographic screening to detect probable effects of electrolyte changes such as negative T-waves due to hyperkalemia were not performed; therefore, clinically inapparent changes could have been overlooked and underestimated. 

Despite broad assumptions, this investigation did not detect a higher risk for CVE or postoperative bleeding in dialysis-dependent patients undergoing PTX and receiving UHD on the day of surgery due to perioperative hyperkalemia.

## 5. Conclusions

The present findings did not show an enhanced complication rate in patients with hyperkalemia or urgent hemodialysis on the day of surgery undergoing parathyroidectomy due to secondary hyperparathyroidism. For the first time, female gender and obesity seemed to affect changes in serum potassium levels or the need for urgent hemodialysis. Physicians should aim for normal serum calcium levels, prevent the incidence of hungry bone syndrome and perform strict inspections of serum potassium levels perioperatively. Enforced dialysis on the day of parathyroidectomy is recommended—rather early and preventatively before complications occur.

## Figures and Tables

**Table 1:** Demographic parameters, comorbidities and details concerning dialysis-dependency comparing both centers.

		Total (*n* = 251)	Berlin (*n* = 130)	Neuss (*n* = 121)	*p*-Value
Sex	male	131 (52%)	75 (58%)	56 (46%)	0.071
	female	120 (48%)	55 (42%)	65 (54%)	
Age (years)	mean ± SD	52 ± 14	52 ± 14	53 ± 13	0.646
Diabetes	yes	59 (24%)	31 (24%)	28 (23%)	0.868
Obesity	yes	77 (31%)	15 (12%)	62 (51%)	<0.001
CVD	yes	70 (28%)	35 (27%)	35 (29%)	0.752
Dialysis-dependency	yes	240 (96%)	126 (97%)	114 (94%)	0.842
Dialysis-depending time (years)	median, range	4.9 (0.2–27)	4.4 (0.2–27)	4 (1–20)	0.235
Kidney Disease	Glomerulonephritis	51 (20%)	35 (27%)	16 (13%)	0.016
	Tubulo-interstitial	38 (15%)	19 (15%)	19 (16%)	
	Hypertensive	35 (14%)	20 (15%)	15 (12%)	
	Diabetic	32 (13%)	18 (14%)	14 (12%)	
	Polycystic	30 (12%)	15 (12%)	15 (12%)	
	Atrophic	14 (6%)	4 (3%)	10 (8%)	
	Alport-Syndrome	4 (2%)	2 (1%)	2 (2%)	
	Amyloidosis	2 (1%)	1 (<1%)	1 (<1%)	
	Other	28 (11%)	13 (10%)	15 (12%)	
	Unknown	17 (7%)	3 (2%)	14 (12%)	
Cinacalcet	yes	128 (51%)	52 (40%)	76 (63%)	<0.001

Values as numbers and percentage or in means ± standard deviation (SD) and median with range. CVD, cardiovascular disease.

**Table 2:** Perioperative parameters and laboratory chemistry comparing both centers.

		Total (*n* = 251)	Berlin (*n* = 130)	Neuss (*n* = 121)	*p*-Value
Operation Procedure	Total PTX	73 (29%)	29 (22%)	44 (36%)	0.028
	Subtotal PTX	159 (63%)	88 (68%)	71 (59%)	
	Selective PTX	19 (8%)	13 (10%)	6 (5%)	
Duration of surgery (min)	mean ± SD	120 ± 39	129 ± 40	113 ± 38	0.010
PTH preop (ng/L)	median, range	849 (85–3108)	612 (85–2164)	1105 (137–3108)	<0.001
PTH postop (ng/L)	median, range	15 (3–1019)	11 (3–440)	23 (4–1019)	<0.001
Ca^2+^ preop (mmol/L)	mean ± SD	2.4 ± 0.24	2.3 ± 0.23	2.4 ± 0.25	0.007
Ca^2+^ min (mmol/L)	mean ± SD	1.8 ± 0.29	1.87 ± 0.27	1.79 ± 0.3	0.025
Hyper K^+^ preop	yes	70 (28%)	30 (23%)	40 (33%)	0.098
Hyper K^+^ intraop	yes	69 (27%)	22 (17%)	47 (39%)	<0.001
Hyper K^+^ postop	yes	74 (29%)	18 (14%)	56 (46%)	<0.001
CVE periop	yes	3 (1%)	2 (2%)	1 (1%)	-
Dialysis at day of surgery	yes	67 (27%)	25 (19%)	42 (35%)	0.006

Values as numbers and percentage, as means ± standard deviation (SD) or as median with range. Ca^2+^, calcium; CVE, cardiovascular event; Hyper K^+^, hyperkalemia; intraop, intraoperative; min, minimum measured; PTH, parathyroid hormone; PTX, parathyroidectomy; periop, perioperative; preop, preoperative; postop, postoperative.

**Table 3:** Comparison of demographic parameters and occurrence of perioperative hyperkalemia between urgent hemodialysis (UHD) recipients (group A) and control B (no dialysis).

		Group A UHD (*n* = 67)	Group B No Dialysis (*n* = 184)	*p*-Value
Hospital	Neuss	42 (63%)	79 (43%)	0.006
	Berlin	25 (37%)	105 (57%)	
Sex	male	27 (40%)	104 (57%)	0.023
	female	40 (60%)	80 (43%)	
Age (years)	mean ± SD	48.8 ± 13.2	51.8 ± 13.8	0.118
Diabetes	yes	16 (24%)	43 (24%)	0.950
Obesity	yes	27 (40%)	50 (28%)	0.040
CVD	yes	20 (30%)	50 (28%)	0.693
Dialysis-depending time (years)	median, range	3.5 (0.2–20)	4.1 (0.4–27)	0.686
Cinacalcet	yes	32 (48%)	96 (52%)	0.815
Duration of surgery (minutes)	mean ± SD	125 ± 36	125 ± 41	0.987
Hyper K^+^ preop	yes	19 (28%)	51 (28%)	0.984
Hyper K^+^ intraop	yes	35 (52%)	34 (18%)	<0.001
Hyper K^+^ postop	yes	36 (54%)	38 (20%)	<0.001

Values as numbers and percentage or in means ± standard deviation (SD) and median with range. CVD, cardiovascular disease; Hyper K^+^, hyperkalemia; intraop, intraoperative; preop, preoperative; postop, postoperative.

**Table 4:** Development of median serum potassium levels (SPL) comparing need for urgent hemodialysis (UHD) and the study centers.

		Group A UHD (*n* = 67)			Group B No Dialysis (*n* = 184)		
		Berlin (*n* = 25)	Neuss (*n* = 42)	*p*-Value	Berlin (*n* = 105)	Neuss (*n* = 79)	*p*-Value
ΔK^+^ preop-intraop (mmol/L)	mean ± SD	+0.1 ± 0.9	+0.6 ± 1.2	0.046	−0.2 ± 0.9	−0.1 ± 0.9	0.927
ΔK^+^ intra-postop (mmol/L)	mean ± SD	−0.3 ± 0.9	+0.1 ± 0.9	0.085	+0.1 ± 0.8	−0.1 ± 0.5	0.462
ΔK^+^ preop-postop (mmol/L)	mean ± SD	−0.2 ± 0.8	+0.7 ± 1.1	<0.001	−0.1 ± 0.9	−0.1 ± 0.9	0.714

Values in means ± standard deviation (SD). Δ, difference; K^+^, serum potassium; intraop, intraoperative; preop, preoperative; postop, postoperative; UHD, urgent hemodialysis.

## Supplementary Materials

The following supporting information can be downloaded at: https://www.mdpi.com/article/10.3390/jcm11020409/s1. Figure S1. Boxplots showing the distribution of SPL at the different times perioperatively with regard for the need of UHD in total and comparing the centers. Figure S2. ROC-analysis of serum potassium levels (SPL) and its predictivity for urgent hemodialysis (UHD) at day of surgery in total and at both centers. The left figure presents the analyses of the whole study group, the middle figure shows the analyses of patients in Berlin and the right figure in Neuss. The blue line represents preoperative SPL, the red line the intraoperative values and the green line shows postoperatively measured SPL. Figure S3. Hypothesis of the pathophysiology of hyperkalemia in patients undergoing PTX due to sharp decrease of SPL resulting from sudden calcium-uptake by the bones. Table S1. Comparison of different serum potassium thresholds intra- and postoperative with regard for urgent hemodialysis (UHD) at both study centers.
