# Supplementary material for: Hyperkalemia Following Parathyroidectomy in Patients with Renal Hyperparathyroidism—New Thresholds for Urgent Perioperative Dialysis
Source: J Clin Med. 2022 Jan 14;11(2):409. doi: 10.3390/jcm11020409 (PMC8777922; doi:10.3390/jcm11020409)
Supplement: Supplementary file 1 [file jcm-11-00409-s001.zip › Figure S1 (SUPPLEMENTARY).pdf]

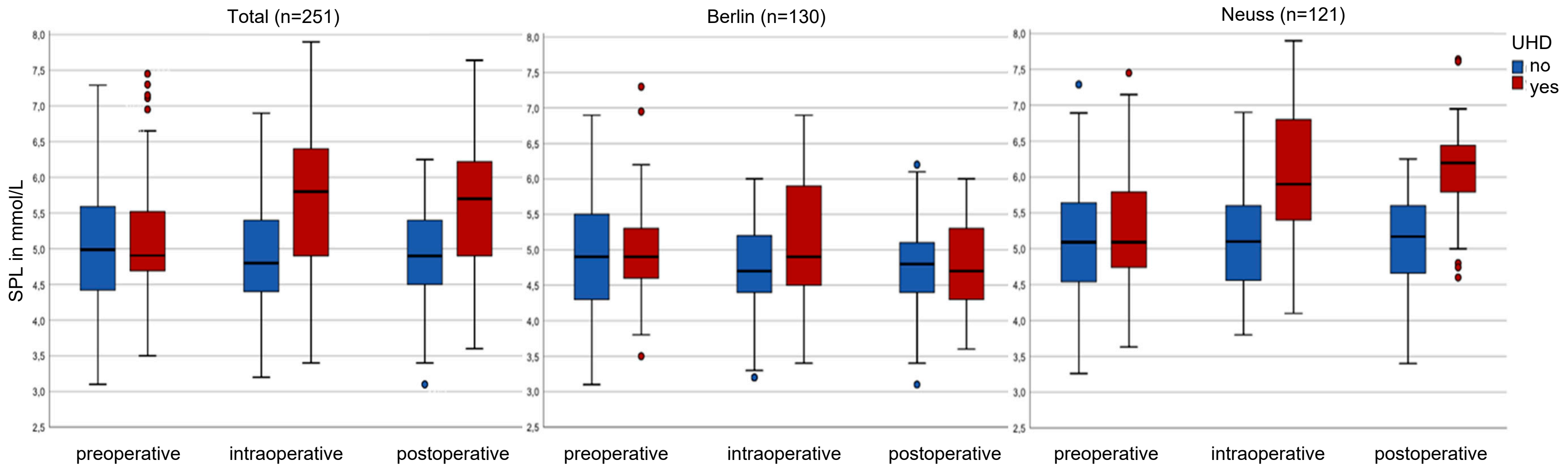

Figure S1 Boxplots showing the distribution of SPL at the different times perioperatively with regard for the need of UHD in total and comparing the centers.
