# Supplementary material for: Hyperkalemia Following Parathyroidectomy in Patients with Renal Hyperparathyroidism—New Thresholds for Urgent Perioperative Dialysis
Source: J Clin Med. 2022 Jan 14;11(2):409. doi: 10.3390/jcm11020409 (PMC8777922; doi:10.3390/jcm11020409)
Supplement: Supplementary file 1 [file jcm-11-00409-s001.zip › Figure S2 (SUPPLEMENTARY).pdf]

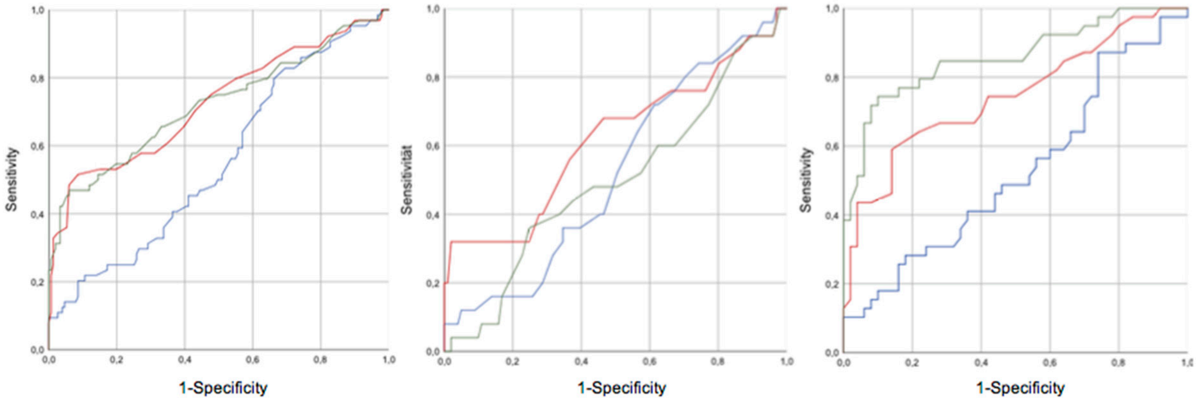

Total (n = 251)					
**Times of measure**	**SPL**	**AUC**	**Sign.**	**Sens.**	**Spec.**
Preoperatively	4.63 mmol/L	0.555	0.204	0.797	0.338
Intraoperatively	5.75 mmol/L	0.725	<0.001	0.516	0.914
Postoperatively	5.83 mmol/L	0.721	<0.001	0.470	0.940
**Charité (n = 130)**					
Preoperatively	4.69 mmol/L	0.521	0.748	0.720	0.386
Intraoperatively	5.80 mmol/L	0.618	0.070	0.320	0.980
Postoperatively	5.15 mmol/L	0.498	0.066	0.360	0.752
**Neuss (n = 121)**					
Preoperatively	4.59 mmol/L	0.525	0.682	0.872	0.260
Intraoperatively	5.85 mmol/L	0.745	0.050	0.590	0.860
Postoperatively	5.83 mmol/L	0.858	0.042	0.744	0.900

SPL serum potassium level, AUC area under the curve, Sign. significance, Spec. specificity

Figure S2 ROC-analysis of serum potassium levels (SPL) and its predictivity for urgent hemodialysis (UHD) at day of surgery in total and at both centers. The left figure presents the analyses of the whole study group, the middle figure shows the analyses of patients in Berlin and the right figure in Neuss. The blue line represents preoperative SPL, the red line the intraoperative values and the green line shows postoperatively measured SPL.
