# Supplementary material for: Hyperkalemia Following Parathyroidectomy in Patients with Renal Hyperparathyroidism—New Thresholds for Urgent Perioperative Dialysis
Source: J Clin Med. 2022 Jan 14;11(2):409. doi: 10.3390/jcm11020409 (PMC8777922; doi:10.3390/jcm11020409)
Supplement: Supplementary file 1 [file jcm-11-00409-s001.zip › Figure S3 (SUPPLEMENTARY).pdf]

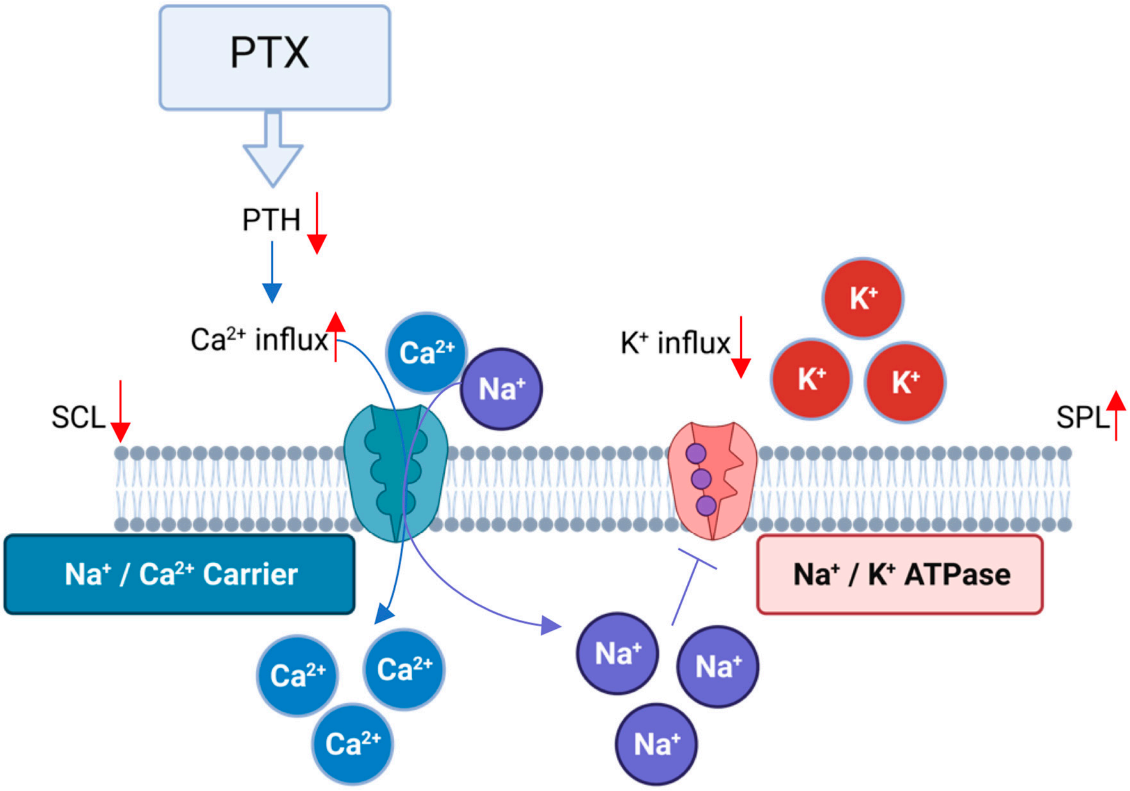

Figure S3 Hypothesis of the pathophysiology of hyperkalemia in patients undergoing PTX due to sharp decrease of SPL resulting from sudden calcium-uptake by the bones.
