# Supplementary material for: Hyperkalemia Following Parathyroidectomy in Patients with Renal Hyperparathyroidism—New Thresholds for Urgent Perioperative Dialysis
Source: J Clin Med. 2022 Jan 14;11(2):409. doi: 10.3390/jcm11020409 (PMC8777922; doi:10.3390/jcm11020409)
Supplement: Supplementary file 1 [file jcm-11-00409-s001.zip › Table S1 (SUPPLEMENTARY).pdf]

**Table S1**	Comparison of different serum potassium thresholds intra- and postoperative with regard for urgent hemodialysis (UHD) at the both study centers.						
		**Berlin** (n = 130)			**Neuss** (n = 121)		
		**UHD** (n = 25)	**No Dialysis** (n = 105)	***p*-value**	**UHD** (n = 42)	**No Dialysis** (n = 79)	***p*-value**
K^+ intraop ≥ 5.5 mmol/L	yes	8 (32%)	14 (13%)	0.032	27 (64%)	20 (25%)	0.001
K^+ intraop ≥ 5.75 mmol/L	yes	8 (32%)	2 (2%)	<0.001	25 (60%)	11(14%)	<0.001
K^+ postop ≥ 5.5 mmol/L	yes	2 (8%)	16 (15%)	0.316	34 (81%)	22 (28%)	<0.001
K^+ postop ≥ 5.75 mmol/L	yes	1 (4%)	10 (10%)	0.349	30 (71%)	8 (10%)	<0.001

Values as numbers and percentage

K^+ serum potassium, intraop intraoperative, postop postoperative, UHD urgent hemodialysis
